# The placental transcriptome of the first-trimester placenta is affected by in vitro fertilization and embryo transfer

**DOI:** 10.1186/s12958-019-0494-7

**Published:** 2019-07-01

**Authors:** Liang Zhao, Xiuli Zheng, Jingfang Liu, Rong Zheng, Rui Yang, Ying Wang, Lifang Sun

**Affiliations:** 1 Department of Obstetrics and Gynecology, Beijing Jishuitan, Hospital, No. 31, Xinjiekou East Street, Xicheng District, Beijing, 100035 People’s Republic of China; 2 Reproductive Medical Center, Department of Obstetrics and Gynecology, Peking University Third Hospital, No. 49, Huayuan North Road, Haidian District, Beijing, 100191 People’s Republic of China

**Keywords:** Placenta, In vitro fertilization and embryo transfer (IVF-ET), Gene expression, Human, Adverse perinatal outcomes

## Abstract

**Background:**

The placenta is a highly specialized temporary organ that is related to fetal development and pregnancy outcomes, and epidemiological data demonstrate an increased risk of placental abnormality after in vitro fertilization and embryo transfer (IVF-ET).

**Methods:**

This study examines alterations in the transcriptome profile of first-trimester placentas from IVF-ET pregnancies and analyzes the potential mechanisms that play a role in the adverse perinatal outcomes associated with IVF-ET procedures. Four human placental villi from first-trimester samples were obtained through fetal bud aspiration from patients subjected to IVF-ET due to oviductal factors. An additional four control human placental villi were derived from a group of subjects who spontaneously conceived a twin pregnancy. We analyzed their transcriptomes by microarray. Then, RT-qPCR and immunohistochemistry were utilized to analyze several dysregulated genes to validate the microarray results. Biological functions and pathways were analyzed with bioinformatics tools.

**Results:**

A total of 3405 differentially regulated genes were identified as significantly dysregulated (> 2-fold change; *P* < 0.05) in the IVF-ET placenta in the first trimester: 1910 upregulated and 1495 downregulated genes. Functional enrichment analysis of the differentially regulated genes demonstrated that the genes were involved in more than 50 biological processes and pathways that have been shown to play important roles in the first trimester in vivo. These pathways can be clustered into coagulation cascades, immune response, transmembrane signaling, metabolism, cell cycle, stress control, invasion and vascularization. Nearly the same number of up- and downregulated genes participate in the same biological processes related to placental development and maintenance. Procedures utilized in IVF-ET altered the expression of first-trimester placental genes that are critical to these biological processes and triggered a compensatory mechanism during early implantation in vivo.

**Conclusion:**

These data provide a potential basis for further analysis of the higher frequency of adverse perinatal outcomes following IVF-ET, with the ultimate goal of developing safer IVF-ET protocols.

**Electronic supplementary material:**

The online version of this article (10.1186/s12958-019-0494-7) contains supplementary material, which is available to authorized users.

## Background

The widespread application of therapeutic methods for subfertile patients, particularly in vitro fertilization and embryo transfer (IVF-ET), has remarkably increased the pregnancy rate [1]. The elective frozen-thawed single-blastocyst transfer approach, which has been widely adopted in multiple reproductive centers, has largely overcome the risks of multiple pregnancies [2, 3]. Recent census results show that the perinatal outcomes of the most recent groups of patients are better than those of previous groups of patients [4]. This positive trend is due to more appropriate embryo transfer strategies, milder ovarian stimulation, laboratory technological advances, and improved culture media [5]. However, even in singleton pregnancy, after adjustment for maternal confounding factors, the risk of multiple adverse outcomes during the perinatal period, including miscarriage, preterm birth, small for gestational age, low birth weight and gestational hypertension, are higher in IVF-ET pregnancies than in spontaneously conceived pregnancies [6–10].

The placenta, derived from the embryonic trophectoderm, is a highly specialized and adaptive temporary organ and is critical for embryonic development and perinatal outcomes [11]. Some animal experiments have shown that placental tissue is more sensitive to external disturbances than embryonic tissue [12, 13]. The possible explanation for this result is twofold. On the one hand, the trophectoderm, which subsequently develops into the placenta, is the first differentiated cell lineage of the embryo to address various changes in the external environment [14]. On the other hand, the trophectoderm, whose genomic imprints are unstable and vulnerable to the surrounding microenvironment during implantation, appears to be sensitive to in vitro culture conditions and prone to an imbalance in imprinted gene expression [15, 16]. Although the composition of the culture medium has continuously been improved, it is still not completely equivalent to the physiological conditions of the natural environment in vivo [17]. In addition, even with careful and gentle manipulation, the trophectoderm must withstand severe stress and interference from the environment, such as changes in temperature, pH, and oxygen tension; light exposure; pH variations during manipulation; and shear stress linked to repeated pipetting, which may interact and synergistically affect placental function and development [18–20].

Professionally, there is an urgent need to understand and determine the impact of IVF-ET on placental development as soon as possible. Although several well-designed studies, which are limited in terms of research design and material acquisition, have focused on the effects of IVF-ET on human placental function and development, these studies used term placentas. These data support the hypothesis that altered global gene expression leads to overrepresented biological pathways in the human term placenta after IVF-ET [21, 22]. Similarly, term and late-gestation placentas have been examined in animal models, and genome-wide mRNA expression revealed that IVF-ET significantly affects placental functions and triggers compensatory responses [23–25]. Due to alterations in the peri-implantation environment and to key functions in placental development, such as cell fusion, embryo implantation, immune tolerance, and tissue remodeling, investigating the influence of IVF-ET on first-trimester placental development and function is particularly important [26, 27]. In addition, according to maternal gestational physiology and the theory of fetal origins of adult disease [28, 29], related research is needed to determine IVF-ET effects on placental gene expression and biological function as soon as possible, which would contribute toward developing strategies to prevent these diseases.

In this study, we hypothesized that altered global gene expression occurs in placental tissue during the first trimester after IVF-ET compared with the first trimester after spontaneous conception. Specifically, we used a twin-to-singleton selective fetal reduction strategy to obtain first-trimester placentas in vivo. Our previous study using immunohistochemistry showed that IVF-ET affects first-trimester placental development and function [30]. Therefore, we carried out a microarray analysis to investigate the effects of IVF-ET treatment on gene expression and potential biological functions in human first-trimester placentas compared with human first-trimester placentas of spontaneous pregnancies. Furthermore, a better understanding of the placental mechanisms triggered by IVF-ET per se may be of future value in improving the safety of IVF-ET protocols.

## Methods

### Study subjects

From January to October 2018, twin-to-singleton selective fetal reduction was performed in 4 cases in the first trimester after IVF-ET. All patients from fresh embryo transfers underwent standard IVF-ET due to oviductal obstruction. The sperm quality test results of each male were normal. In this study, the reason for fetal reduction from twin to singleton was patient preference. The application of the IVF-ET clinical technology was licensed and authorized by the National Health Commission of the People’s Republic of China. The control group included 4 cases of unwanted spontaneously conceived monochorionic twins in the same period. Clinical data were collected at the obstetrics outpatient clinic at Beijing Jishuitan Hospital and organized in a database. The number of specimens needed for the analysis of variance in the microarray experiment, which was 4, was determined by the study’s statistical criteria. Information about the couple’s weights, smoking habits, alcohol consumption habits and hormone use was collected by questionnaire. Information on maternal age, gestational weeks, gravidity history and body mass index (BMI) was matched between the IVF-ET and spontaneously conceived groups.

### Sample collection and ethics

For all cases in which a singleton was retained from a twin pregnancy, reductions were performed by the same senior physician by fetal bud aspiration under ultrasound guidance. We selected 4 cases for villi suction simultaneously. We used a special needle that was authorized by the State Intellectual Property Office of People’s Republic of China (No ZL 2015 20,035,543.7). The placental tissue was collected 30–35 days after embryo transfer, which is equivalent to 7–8 gestational weeks. The pregnancies in the control group of double chorionic twin pregnancies were diagnosed with early intrauterine pregnancy based on urine pregnancy tests and transvaginal ultrasound assessments. None of the patients had taken steroid hormone drugs for the previous 3 months and had a record of regular menstruation. The villi were collected during the conventional artificial abortion operation. The villi samples were isolated and purified immediately from the specimens according to the morphology within 30 min under an inverted microscope. All placental tissues were refloated with ice-cold PBS and then stored under RNase-free conditions at − 80 °C until future tissue homogenization and total RNA extraction. Another portion of the remaining specimens was transferred to 4% paraformaldehyde within 30 min after operation and incubated at 4 °C overnight. Then, the specimens were stored in 1% paraformaldehyde at 4 °C until routine dehydration and embedding in paraffin. All study participants were enrolled at the Beijing Jishuitan Hospital 2018, were of Asian ancestry and were living in Beijing.

### RNA extraction

Tissue homogenization and total RNA extraction were accomplished at CapitalBio Corporation, Bejing, China, using the Macherey–Nagel NucleoSpin RNA II kit (Macherey–Nagel, Duren, Germany), where a significant reduction in genomic DNA and protein contamination was achieved. Total RNA was derived from approximately 200 mg of first-trimester placental tissue, including various placental types. A NanoDrop ND100 spectrophotometer (NanoDrop Technologies, USA) was used to evaluate the purity and concentration of isolated total RNAs. The intensity ratio of ribosomal 28S and 18S RNA was 1.5–1.8:1. The RNA was used in both the microarray assay and subsequent RT-qPCR analysis.

### Microarray hybridization

Thirty nanograms of total RNA was amplified and transcribed into biotinylated cRNA according to the instructions provided in the MessageAmp II aRNA Amplification Kit (Ambion Inc., Carlsbad, Calif., USA). Samples of cDNA were hybridized to the Affymetrix Human Genome U133 Plus 2.0 Array. The cDNA of each sample was hybridized to one Array. This array contains approximately 54,000 oligonucleotide probes and covers more than 30,000 human genes mapped through UniGene or via RefSeq annotation (Affymetrix, Santa Clara, CA, USA). Then, the arrays were washed and stained on the Fluidics Station 450 (Affymetrix) and scanned on an Affymetrix GeneChip Scanner 3000 (Affymetrix) to analyze the hybridization data according to the manufacturer’s instructions. The data were collected from the scanned images and evaluated using the Affymetrix GeneChip Operating Software (GCOS 1.4). Differentially expressed genes between the IVF-ET and control groups were analyzed using Significance Analysis of Microarrays (SAM version 3.02, Stanford University, Stanford, CA).

### Microarray data analysis

Our primary microarray data for the first-trimester placental samples have been submitted to the Gene Expression Omnibus of the National Center for Biotechnology Information [31]. The data can be obtained through GEO Series accession number GSE 122214 (https://www.ncbi.nlm.nih.gov/geo/query/acc.cgi?acc=GSE122214). Data quality, linear modeling and significance analysis were performed by means of Bioconductor package linear models [32]. The cut-off value was determined to be a twofold difference in expression values. The proportion of false-positive corrections of < 0.05 was considered statistically significant. If a gene had multiple probe identification data in the results, the average fold change was calculated.

To assess the variability in placental tissues with nonparametric methods, hierarchical clustering and principal component analysis were performed to visualize global variation. Based on the difference in placental gene expression between IVF-ET pregnancy and spontaneously conceived pregnancy, a three-dimensional scatter plot was used to visualize the sample gene variation in the principal component analysis. The distance between different points was calculated using the number and covariance of differentially expressed genes. The complete-linkage hierarchical clustering algorithm was used to assess the variation among gene expression profiles.

Enrichment and integrated analysis of differentially regulated genes were performed by submitting data to Database for Annotation, Visualization and Integrated Discovery (DAVID), Ingenuity Pathway Analysis (IPA), Kyoko Encyclopedia of Genes and Genomes (KEGG), and KEGG Orthology Based Annotation System (KOBAS). According to Gene Ontology terms, DAVID divided blocks of functionally related differentially expressed genes based on biological process, molecular function and cellular component. The other three databases were used to visually integrate dysregulated genes and identify statistically significant enriched pathways. Up- and downregulated genes were investigated separately. Statistical controls were based on all human genes included in the Affymetrix Human Genome U133 Plus 2.0 Array. A false discovery rate (FDR) < 0.05 and *p*-value < 0.05 were used to determine statistical significance.

### Quantitative real-time RT-PCR

Validation of the microarray results was performed by real-time RT-qPCR using the same samples for microarray. Synthesis of cDNA was achieved using the PrimeScript RT Reagent Kit (TaKaRa Biotechnology, Dalian, China) with 2 μg of RNA. RT-qPCR was performed with an ABI Prism 7300 Sequence Detection System (Applied Biosystems, Roche, USA) according to recommendations of the manufacturer. The following genes were used for microarray validation: alpha-fetoprotein (AFP); glial cells missing homolog 1 (Drosophila) (GCM1); leukocyte-associated immunoglobulin-like receptor 2 (LAIR2); phosphatase and tension homolog (PTEN); B-cell CLL/lymphoma 2 (BCL2); transferrin (TF); metallothionein 1 M (MT1M); epidermal growth factor receptor (EGFR); tubulin, beta 1 class VI (TUBB1); and vascular endothelial growth factor (VEGF). Considering their critical functions in the first-trimester placenta based on the biological analysis, these ten target genes, which showed differential expression in the microarray analysis, were selected and tested by RT-qPCR. Three technical and five biological replicates were run for all RT-qPCR analyses. Relative mRNA expression levels were calculated using the comparative threshold ΔΔCt method. Comparison of gene expression between the IVF-ET and control groups was performed using a two-tailed Student’s *t*-test to determine significant differences. *P* < 0.05 was considered significant. A list of all predesigned PCR primer pairs used in the RT-qPCR analysis is provided (Additional file 1: Table S1).

### Immunohistochemistry (IHC)

The first-trimester placental tissues used for immunohistochemical staining originated from the same tissues used for the previous RNA extractions. The placental tissues that were stored in PFA/PBS were fixed and embedded in paraffin. Then, these samples were deparaffinized, rehydrated and sectioned. Immunohistochemical localization was performed to analyze the AFP, VEGF, TF, TUBB1, BCL2, GCM1, PTEN and LAIR2 gene products using 5-μm paraffin sections mounted on Superfrost Plus slides (Gerhard Menzel, Braunschweig, Germany). The primary antibodies utilized were as follows: AFP (1:25 dilution, ZM-0009; ZSGB-Bio, Beijing, China), VEGF (1:25 dilution, ZA0509, ZSGB-Bio, Beijing, China), TF (1:25 dilution, TA500848, ZSGB-Bio, Beijing, China), TUBB1 (1:25 dilution, TA506588, ZSGB-Bio, Beijing, China), BCL2 (1:25 dilution, ZA-0010; ZSGB-Bio, Beijing, China), GCM1 (1:100 dilution, ab187860; Abcam, Cambridge, UK), PTEN ((1:25 dilution, ZA-0635; ZSGB-Bio, Beijing, China), and LAIR2 (1:100 dilution, ab183145, Abcam, Cambridge, UK). Protein detection and negative control assessment were conducted simultaneously following the manufacturer’s instructions. A microscope magnification of × 400 was used. Two senior pathologists examined the stained sections in a double-blinded fashion and performed the quantifications manually. The positivity rates are described as stained cells vs the total number of cells.

## Results

### Characteristics of the patients

In terms of age, gestational age, parity and BMI, there were no significant differences between the women who were subjected to IVF-ET and those who spontaneously conceived (Table 1).

**Table 1:** Characteristics of the patients in the two groups

	N	Mean age	Gestational age (day)	Parity	BMI (kg/m2)
IVF-ET group	4	30.66 ± 3.76	49.44 ± 3.14	1.10 ± 1.21	23.33 ± 3.17
Control group	4	28.86 ± 3.45	49.35 ± 3.23	2.21 ± 0.89	22.99 ± 2.35
t		0.833	0.637	0.858	1.311
p		0.425	0.529	0.437	0.263

### Microarray data

**Fig. 1:**
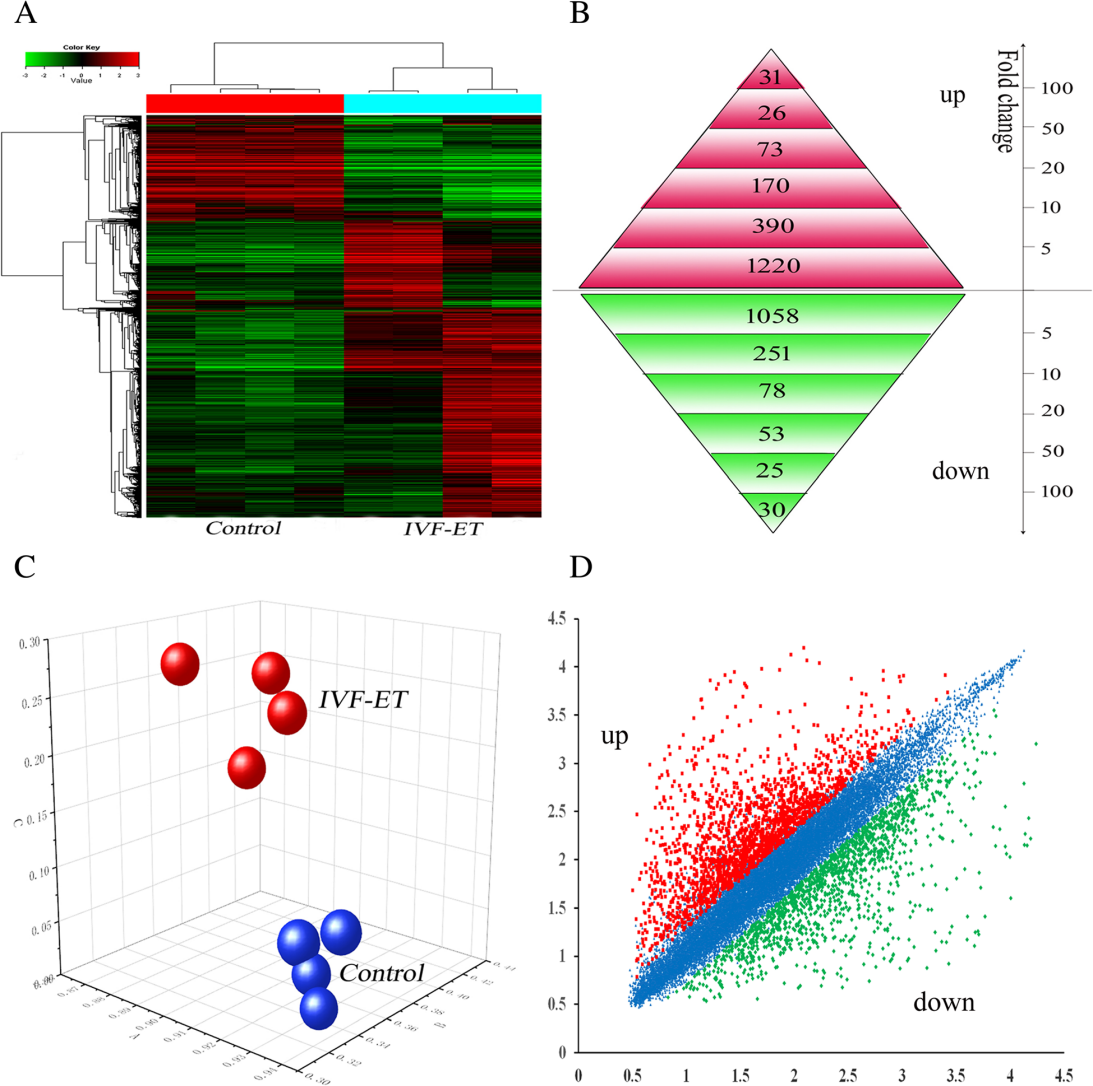
Supervised hierarchical clustering and principal component analysis of the first-trimester placenta from the IVF-ET and control groups. **a** Supervised hierarchical clustering was performed with 3405 differentially expressed genes between the IVF-ET and naturally conceived pregnancies, allowing the distinction of the two sample groups. **b** Number of genes specifically modulated during the first trimester in the placenta after IVF-ET. **c** Principal component analysis performed on the placenta after IVF-ET and naturally conceived pregnancy in the first trimester based on the results of the microarray analysis. **b**: IVF-ET; N: Control). (**d**) Examples of the graphic display of expression profiling data obtained from the microarray analysis. The vertical axis is the log fold change in gene expression

Transcriptomic profiling of 8 human first-trimester placental samples was performed on samples obtained from 4 IVF-ET and 4 spontaneously conceived pregnancies. A total of 3405 differentially expressed genes were validated according to a false discovery rate of 0.05. A total of 1910 genes were expressed at higher levels, and 1495 genes were expressed at lower levels in the first-trimester placentas produced by IVF-ET compared with those produced by natural conception. Interestingly, the numbers of upregulated (56.1%) and downregulated (43.9%) genes were well balanced (Fig. 1a, b, d).

Hierarchical clustering analysis of the microarray data demonstrated an expressional alteration and separation of placental dysregulated transcripts between the IVF-ET and control groups (Fig. 1a). Alternatively, the principal component analysis was applied to placental gene expression profiles and demonstrated the same separate pattern, where the two study groups clustered into two separate groups (Fig. 1c).

**Fig. 2:**
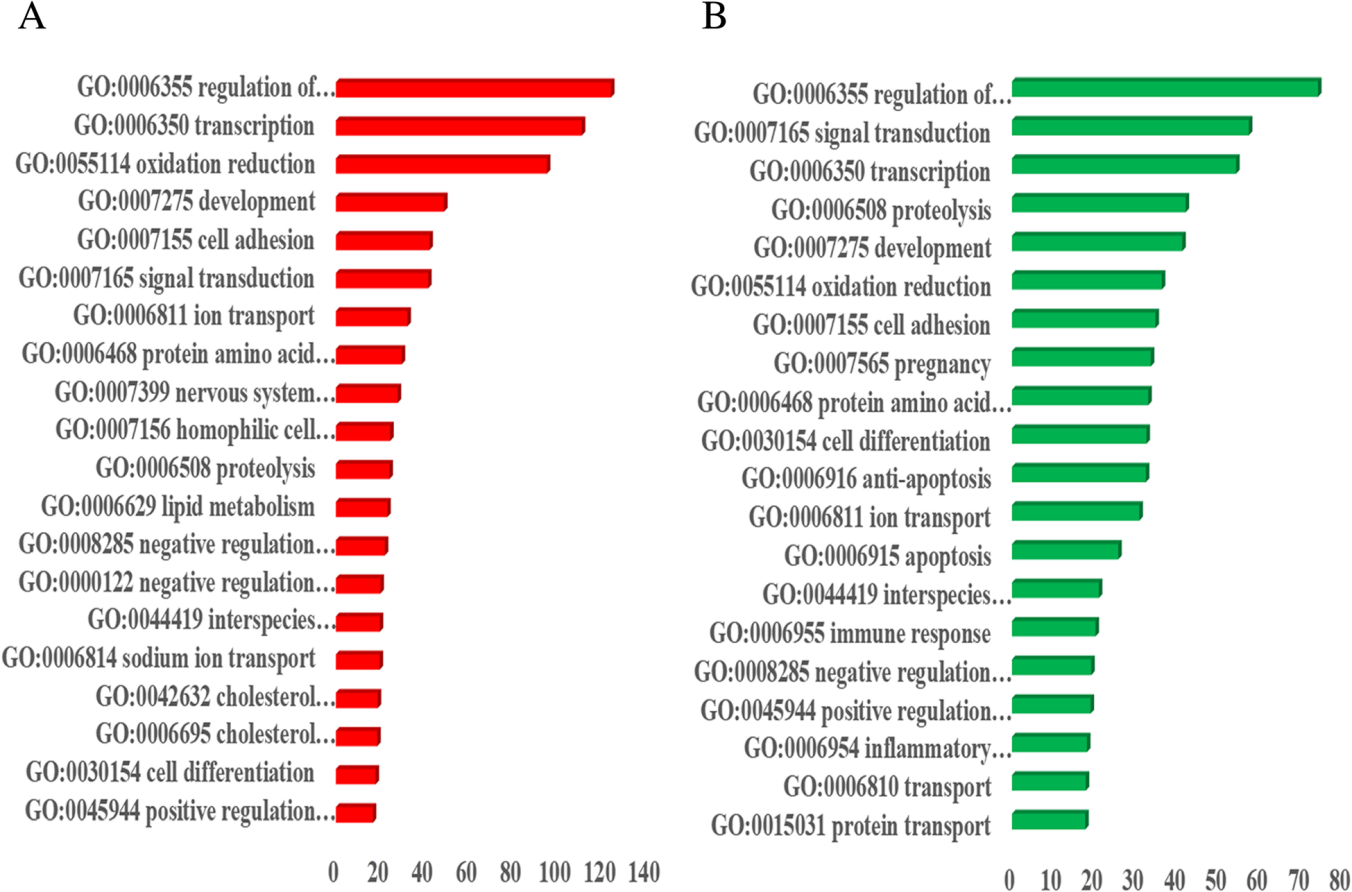
Functional enrichment analysis of differentially regulated genes in the placenta after IVF-ET compared with the control group during the first trimester according to biological processes. Red, upregulated genes; green, downregulated genes

The functional enrichment annotation of differentially expressed genes demonstrated that more than 50 Gene Ontology (GO) categories and pathways were successfully clustered in the list of dysregulated transcripts. Many broad biological processes of upregulated genes in pregnancy progression were regulation of transcription DNA-dependent (*n* = 149, FDR *P* = 1.15 × 10^− 124^), transcription (*n* = 143, FDR *P* = 1.55 × 10^− 111^), oxidation reduction (*n* = 82, FDR *P* = 7.98 × 10^− 96^), development (*n* = 84, FDR *P* = 1.95 × 10^− 49^), and cell adhesion (*n* = 50, FDR *P* = 7.37 × 10^− 43^). The biological processes of the downregulated genes were regulation of transcription DNA-dependent (*n* = 102 FDR *P* = 8.38 × 10^− 75^), signal transduction (*n* = 103, FDR *P* = 4.54 × 10^− 58^), transcription (*n* = 87, FDR *P* = 5.65 × 10^− 55^), proteolysis (*n* = 53, FDR *P* = 8.86 × 10^− 43^), and development (*n* = 72, FDR *P* = 6.00 × 10^− 42^). Additional biological processes for the up- and downregulated genes are listed (Additional file 2: Table S2 Additional file 3: Table S3). Interestingly, almost the same numbers of up- and downregulated genes are allocated in the same biological process related to placental development and maintenance (Fig. 2). These differentially expressed genes were widely distributed to the nucleus, cytoplasm and cell membrane of the placenta. The GO analyses also highlight the disturbances in various gene and molecular functions and their link to pregnancy complications.

**Fig. 3:**
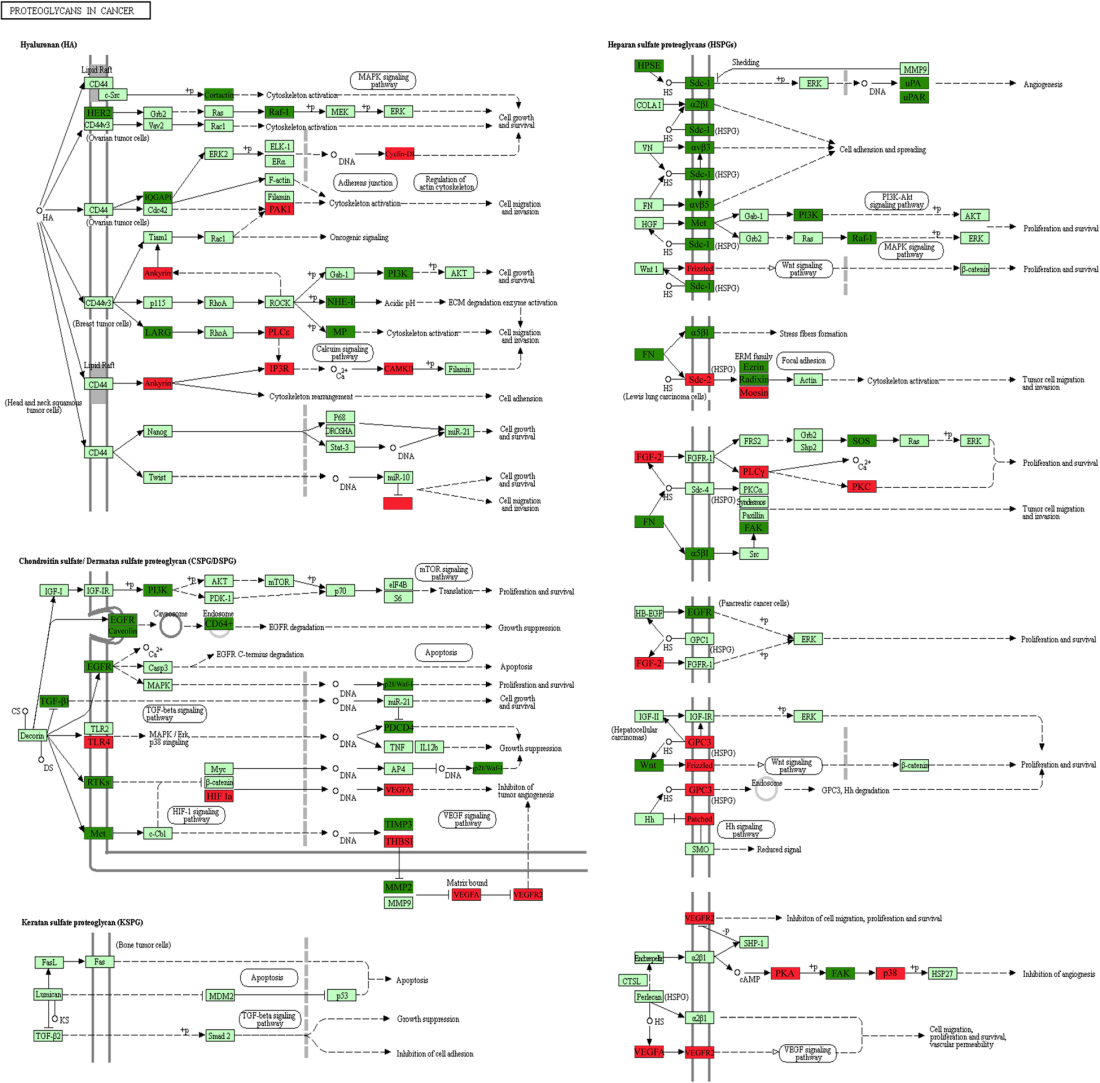
Differentially expressed genes were labeled in the proteoglycans in cancer pathway in the placenta after IVF-ET during the first trimester by KEGG. Red, upregulated genes; Dark green, downregulated genes; Light green, no change

In the IPA, some networks were built based on the biological processes of differentially regulated placental gene expression in the IVF-ET group (Table 2). These abnormal pathways included metabolic pathways (*n* = 279, FDR *P* = 7.43 × 10^− 22^), complement and coagulation cascades (*n* = 44, FDR *P* = 5.74 × 10^− 14^), Proteoglycans in cancer (*n* = 58, FDR *P* = 4.17 × 10^− 8^), the PPAR signaling pathway (*n* = 23, FDR *P* = 1.08 × 10^− 4^), the Wnt signaling pathway (*n* = 35, FDR *P* = 1.53 × 10^− 4^), and the Jak-STAT signaling pathway (*n* = 36, FDR *P* = 5.50 × 10^− 4^), among others. For significant proteoglycans in cancer and the PPAR signaling pathway, we labeled the up- (red) and downregulated (green) genes in the signaling pathways (Fig. 3, Additional file 4: Figure S1).

**Table 2:** Cluster of significant pathways related to IVF-ET

No	Term	ID	Input number	*P*-Value
1	Metabolic pathways	hsa01100	279	7.43 × 10^− 22^
2	Complement and coagulation cascades	hsa04610	44	5.74 × 10^−14^
3	Proteoglycans in cancer	hsa05205	58	4.17 × 10^−8^
4	Pathways in cancer	hsa05200	87	3.72 × 10^−7^
5	Insulin resistance	hsa04931	33	1.08 × 10^−5^
6	Carbon metabolism	hsa01200	33	1.46 × 10^−5^
7	Axon guidance	hsa04360	44	2.23 × 10^−5^
8	Ras signaling pathway	hsa04014	52	3.77 × 10^−5^
9	Glycine, serine and threonine metabolism	hsa00260	17	4.59 × 10^−5^
10	Mineral absorption	hsa04978	19	8.74 × 10^−5^
11	PPAR signaling pathway	hsa03320	23	1.08 × 10^−4^
12	Wnt signaling pathway	hsa04310	35	1.53 × 10^− 4^
13	Transcriptional misregulation in cancer	hsa05202	41	1.72 × 10^−4^
14	AGE-RAGE signaling pathway in diabetic complications	hsa04933	28	1.74 × 10^−4^
15	HIF-1 signaling pathway	hsa04066	28	2.28 × 10^−4^
16	mTOR signaling pathway	hsa04150	36	3.31 × 10^−4^
17	Fatty acid metabolism	hsa01212	17	3.32 × 10^−4^
18	Focal adhesion	hsa04510	44	3.81 × 10^−4^
19	Regulation of actin cytoskeleton	hsa04810	46	3.91 × 10^−4^
20	Fatty acid degradation	hsa00071	16	3.94 × 10^−4^
21	Steroid hormone biosynthesis	hsa00140	19	3.94 × 10^−4^
22	Bile secretion	hsa04976	21	4.29 × 10^−4^
23	Aldosterone-regulated sodium reabsorption	hsa04960	15	4.65 × 10^−4^
24	Jak-STAT signaling pathway	hsa04630	36	5.50 × 10^−4^
25	ErbB signaling pathway	hsa04012	24	6.18 × 10^−4^
26	Steroid biosynthesis	hsa00100	10	6.33 × 10^−4^
27	Valine, leucine and isoleucine degradation	hsa00280	16	7.02 × 10^−4^
28	Renal cell carcinoma	hsa05211	20	7.19 × 10^−4^
29	Biosynthesis of amino acids	hsa01230	21	9.01 × 10^−4^
30	Tyrosine metabolism	hsa00350	13	9.74 × 10^−4^
31	Tryptophan metabolism	hsa00380	14	1.01 × 10^−3^
32	EGFR tyrosine kinase inhibitor resistance	hsa01521	22	1.09 × 10^−3^
33	Cytokine-cytokine receptor interaction	hsa04060	51	1.10 × 10^−3^
34	Arginine biosynthesis	hsa00220	10	1.11 × 10^− 3^
35	Proximal tubule bicarbonate reclamation	hsa04964	10	1.45 × 10^−3^
36	Sphingolipid metabolism	hsa00600	15	1.46 × 10^−3^
37	Rap1 signaling pathway	hsa04015	43	1.48 × 10^−3^
38	Hippo signaling pathway-multiple species	hsa04392	11	1.64 × 10^−3^
39	FoxO signaling pathway	hsa04068	30	1.79 × 10^−3^
40	Glycerolipid metabolism	hsa00561	17	1.84 × 10^−3^
41	Vitamin digestion and absorption	hsa04977	10	1.87 × 10^−3^
42	Pentose phosphate pathway	hsa00030	11	2.05 × 10^−3^
43	cAMP signaling pathway	hsa04024	40	2.13 × 10^−3^
44	AMPK signaling pathway	hsa04152	28	2.26 × 10^−3^
45	Arginine and proline metabolism	hsa00330	15	2.41 × 10^−3^
46	Alanine, aspartate and glutamate metabolism	hsa00250	12	2.60 × 10^−3^
47	Epithelial cell signaling in Helicobacter pylori infection	hsa05120	18	3.00 × 10^−3^
48	beta-Alanine metabolism	hsa00410	11	3.13 × 10^−3^
49	Insulin signaling pathway	hsa04910	30	3.13 × 10^−3^
50	Propanoate metabolism	hsa00640	11	3.81 × 10^−3^

### Quantitative RT-PCR

**Fig. 4:**
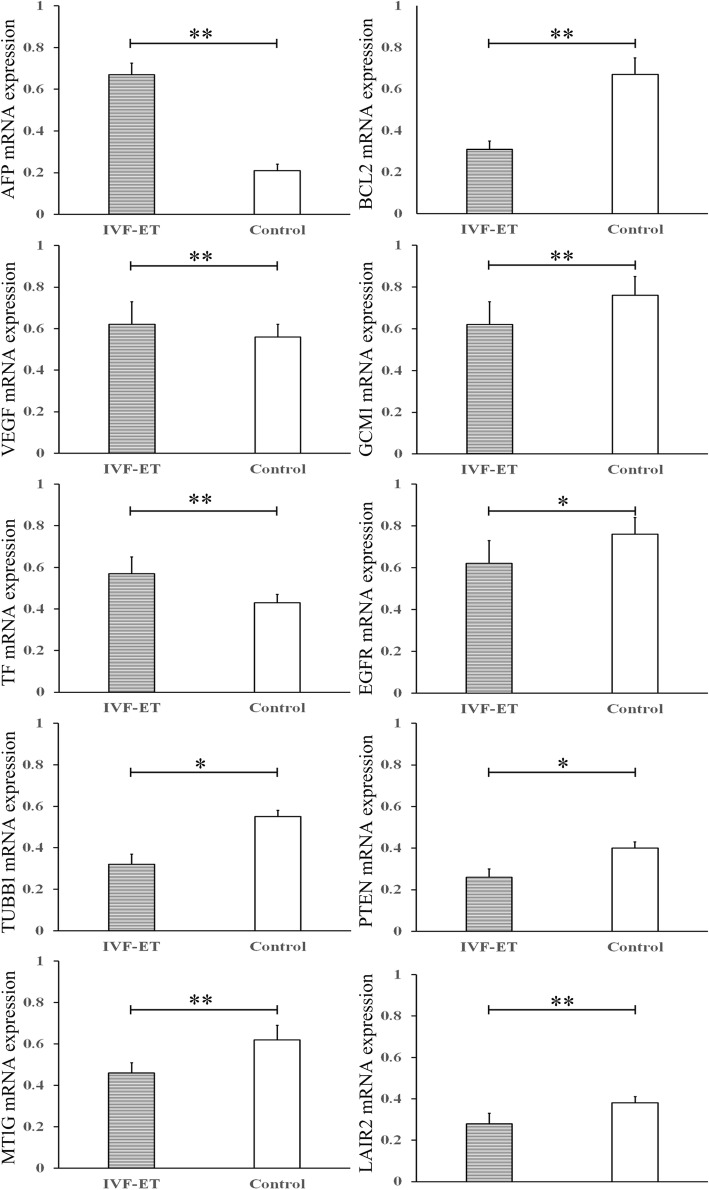
Validation by RT-qPCR for selected genes from first-trimester placentas derived from IVF-ET compared with those derived from the control group. The mRNA levels of AFP, GCM1, LAIR2, PTEN, BCL2, TF, MT1M, EGFR, VEGF, and TUBB1 in the IVF-ET and control groups were examined by RT-qPCR. All data are expressed as the mean ± standard deviation, * indicates *P* < 0.05; ** indicates *P* < 0.01

RT-qPCR analysis of ten selected genes was performed to validate the microarray data. These ten differentially regulated genes were selected according to their biological processes, which were mostly related to placental functions (Fig. 4). The RT-qPCR results confirmed the upregulation the AFP, VEGF, and TF genes and the downregulation of the BCL2, GCM1, EGFR, PTEN, LAIR2, TUBB1,and MT1G genes in the first-trimester placenta after IVF-ET compared with that after natural conception. We observed a significant concordance among the ten tested genes with the array hybridization obtained previously and confirmed more accurate gene expression differences.

### Immunohistochemistry

**Fig. 5:**
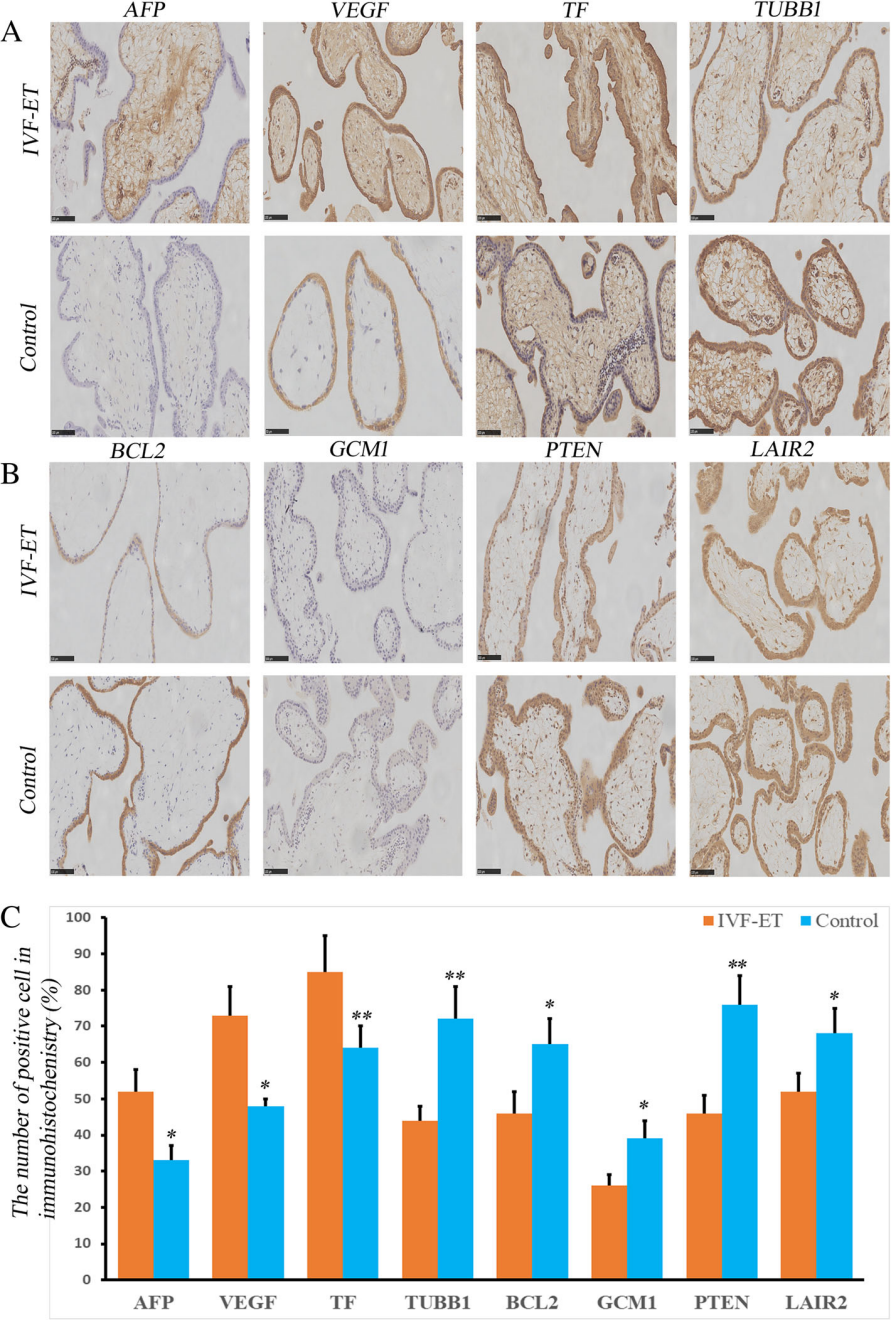
Immunohistochemical analysis of the expression of 8 genes in the first-trimester placental tissues of the IVF-ET and control groups. **a** Immunohistochemical analysis of the expression levels of AFP, VEGF, TF and TUBB1 in the first-trimester placentas of the IVF-ET and control groups. **b** Immunohistochemical analysis of the expression levels of BCL2, GCM1, PTEN and LAIR2 in the first-trimester placentas of the IVF-ET and control groups. **c** Immunohistochemical analysis of the number of positively stained cells expressing the 8 genes in the IVF-ET and control groups. All data are expressed as the mean ± standard deviation, * indicates *P* < 0.05; ** indicates *P* < 0.01, Scale bar = 100 μm

The localizations of the AFP, VEGF, TF, TUBB1, BCL2, GCM1, PTEN and LAIR2 proteins, which represent different biological processes in placentation, were investigated in the human first-trimester placenta (Fig. 5). These 8 genes were mainly localized to the cytoplasm, cell membrane and intercellular spaces of the human first-trimester placenta. In addition, both the IVF-ET group and control group showed expression of these 8 genes. A larger number of cells showed strong AFP, VEGF and TF staining, with cytoplasmic staining, in the first-trimester IVF-ET placental group than in the control group. The control group had higher expression of TUBB1, BCL2, GCM1, PTEN and LAIR2 than the IVF-ET group. The immunohistochemical expression of these genes is consistent with the results of the microarray and RT-qPCR.

## Discussion

Epidemiological studies have demonstrated that even singleton pregnancies conceived after IVF-ET are more likely to develop prematurity, gestational hypertension, placental abruption, low birth weight and intrauterine growth retardation [6–10]. The risk of adverse outcomes originating from suboptimal placentation in pregnancies after IVF-ET is significantly greater than that in spontaneously conceived pregnancies [33, 34]. Several meta-analysis and animal-model studies have demonstrated that this phenomenon is due not only to the infertility diagnosis but also to IVF-ET itself [35–37]. Early placental implantation involves a series of important events, namely, early trophoblast migration, cell fusion, tissue remodeling, immune tolerance, metabolism and extracellular matrix degradation [38–40]. As these trophoblast properties disappear at the end of pregnancy [41, 42], it is particularly critical to study the effects of IVF-ET on first-trimester placental gene expression. Here, we demonstrated that IVF-ET pregnancies show altered expression of first-trimester placental genes that are critical to development and function compared with spontaneously conceived pregnancies. Genome-wide mRNA expression analysis of the first-trimester placenta after IVF-ET also revealed 50 significantly overrepresented biological pathways. Our findings provide evidence of functional placental pathology and a potential basis for understanding the increased risk associated with the first-trimester placenta following IVF-ET.

In the present study, we identified and confirmed that there was a significant difference in first-trimester placental gene expression between IVF-ET and spontaneous pregnancies. We report 3405 differentially expressed genes, including 1910 upregulated and 1495 downregulated genes. These genes were roughly clustered according to their biological functions: cellular process, physiological, biological regulation, metabolism, regulation of biological process, multicellular organismal process, catalytic activity, developmental process, binding and other functions. Our results are consistent with those of most recent studies and support the hypothesis that IVF-ET procedures affect placental gene expression, which subsequently may lead to a risk of adverse perinatal outcomes [43–45]. However, after comparing our data regarding the altered transcriptome in the first-trimester placenta with data from previous studies on the term placenta and endometrium during early implantation, we found that the IVF-ET procedure has a broader and more severe effect on early placentation [21, 22, 46]. Three term placentas from IVF-ET were examined using the same gene chip in our analysis, and only 18 differentially expressed genes and 6 biological functions were reported [21]. Another current study used 10 term placentas from IVF-ET for microarray analysis and identified 1866 differentially expressed genes, including 839 upregulated and 927 downregulated genes [22]. In addition, considering that modification of the maternal endometrium may affect trophoblast invasion and tissue remodeling, 641 differentially expressed genes were identified by microarray analysis from 23 samples from human endometrial biopsies [46]. Our results suggest that early placental tissue may undergo more extensive genetic and functional changes than term placental and other tissues from IVF-ET.

IPA of our data revealed that more than 50 pathways were disrupted in the first-trimester placenta derived from IVF-ET. Further analysis showed that these pathways are widely involved in key events of early placental development and function, whose disruption can lead to adverse outcomes [47–49]. We report the enrichment of multiple genes in the top 50 significantly overrepresented biological pathways. As expected, the first group of pathways comprise metabolic pathways. The IVF-ET process changes the physiological hypoxic environment of the trophoblast in vivo [50]. Metabolic disturbances may lead to metabolic reprogramming of the trophoblast in the first-trimester placenta [51]. Furthermore, the abnormal metabolic characteristics appear to be irreversible, even under normal conditions [52]. Abnormal metabolites, including prostaglandins, leukotrienes, lipid peroxidation and inflammatory mediation, may contribute to vasoconstriction and inflammatory syndrome, eventually resulting in adverse perinatal outcomes [53–55]. Complement and coagulation cascades contribute to homeostasis, thrombosis, defense infection, immune tolerance and apoptotic cell clearance [56, 57]. Abnormalities in these cascades may lead to recurrent pregnancy loss, vascular complications and preeclampsia [58, 59]. The PPAR signaling pathway plays an important role in trophoblast fusion, migration, angiogenesis, lipid metabolism and trophoblast cycle progression [60]. Abnormalities in the PPAR signaling pathway may contribute to increased apoptosis of trophoblasts, trophoblast fusion disorder and necrosis of syncytiotrophoblasts, resulting in severe preeclampsia and intrauterine growth restriction. [61, 62]. However, in most cases involving humans, no immediate abortion has been observed, and most women give birth successfully after IVF-ET, which supports the hypothesis that interference by IVF-ET can trigger a placental compensatory mechanism [63, 64].

Moreover, regarding the compensatory mechanism in the first-trimester placenta after IVF-ET, we analyzed altered gene expression as a whole, rather than at the individual gene level. Interestingly, we separately classified upregulated genes and downregulated genes from IVF-ET sources according to their biological functions and found that the biological functions affected by these genes were consistent between the up- and downregulated genes. In each of the affected biological function categories, the total numbers of upregulated and downregulated genes are similar. From the perspective of signaling pathways, we simultaneously demonstrated and provided the locations of the up- and downregulated genes. Our labeled pathway maps demonstrate that multiple local functional groups are simultaneously affected by the upregulation and downregulation of these genes, resulting in a nearly balanced biological function. The compensatory mechanism in placental development is also observed in animal models. In early pregnancy, the placenta of the IVF-ET mouse pregnancy is smaller than that of the naturally conceived pregnancy [65], and in the third trimester and delivery stage, the placenta is heavier than that of the control [66]. Likewise, even after adjusting for confounding factors in IVF-ET, the human placenta and neonatal weight ratio are often higher than those of the natural control [67]. However, a heavier placenta does not represent more efficient transport of oxygen and nutrients [68]. Another study demonstrated that the weight and size of the placenta is associated with coronary disease and life expectancy [69, 70]. Nevertheless, the human placental compensatory mechanism can only reach a certain point. More severe disruptions can lead to immediate and subsequent adverse outcomes.

To our knowledge, our research is the first to demonstrate alterations in human first-trimester placental gene expression in vivo during early implantation. However, we understand that there are a few limitations with the design of our study. The first limitation is the sample size, but the results were strengthened by the fact that the samples were similar in terms of age, BMI and gestational weeks. The second limitation is twin pregnancy. Although we obtained first-trimester placental tissues in vivo using a fetal reduction strategy, a twin pregnancy has a potential impact on placentation and does not fully reflect a singleton pregnancy. In this study, we focused only on the effect of IVF-ET on the first-trimester placental function and compensatory mechanism in vivo. Additionally, even though the samples were carefully examined under a microscope and stained by immunohistochemistry, we were still concerned about interference from maternal and embryonic cells. Nevertheless, we still believe that these altered gene expression patterns in the first-trimester placenta may partially explain the clinical characteristics and adverse perinatal outcomes of IVF-ET.

## Conclusion

Our study describes the altered gene expression patterns in the early placenta of IVF-ET pregnancies compared to those in spontaneously conceived pregnancies. The abnormal biological functions and pathways associated with these differentially expressed genes provide a potential mechanism for understanding adverse perinatal outcomes following IVF-ET. Our contribution is the verification of a novel hypothesis that the increased risk of adverse perinatal outcomes in IVF-ET originates from early abnormal placentation. The initially disrupted placentation triggers a compensatory mechanism, and fortunately, most of these compensatory actions are successful. The innovation of our study is the examination of the first-trimester human placenta in vivo during the critical periods of implantation in IVF-ET, enabling relevant research to further investigate the target genes and pathways in vivo and not just in animal models. These data provide a potential basis for further analysis of the higher frequency of adverse perinatal outcomes following IVF-ET, with the ultimate goal of developing safer IVF-ET protocols.

## Additional files

Additional file 1: **Table S1.** Detail information on the selection of primers for real-time RT-PCR experiments (DOC 34 kb)

Additional file 2: **Table S2.** The biological processes of up regulated genes in placental subjected to IVF-ET (DOC 68 kb)

Additional file 3: **Table S3.** The biological processes of down regulated genes in placental subjected to IVF-ET (DOC 70 kb)

Additional file 4: **Figure S1.** KEGG analysis of the differentially expressed genes, which are labeled, in the PPAR signaling pathway in the placenta after IVF-ET during the first trimester by KEGG. Red, upregulated; Blue, downregulated; Green, no change. (TIF 14811 kb)

## Data Availability

The primary microarray data of the placental samples have been submitted to the Gene Expression Omnibus of the National Center for Biotechnology Information. The data can be obtained through GEO Series accession number GSE 122214 (https://www.ncbi.nlm.nih.gov/geo/query/acc.cgi?acc=GSE122214). Other data generated or analyzed during this study are available from the corresponding author upon reasonable request.

## Abbreviations

AFP: Alpha-fetoprotein
BCL2: B-cell CLL/lymphoma 2
BMI: Body mass index
DAVID: Database for annotation, visualization and integrated discovery
EGFR: Epidermal growth factor receptor
FDR: False discovery rate
GCM1: Glial cells missing homolog 1 (Drosophila)
IHC: Immunohistochemistry
IPA: Ingenuity pathway analysis
IVF-ET: In vitro fertilization and embryo transfer
KEGG: Kyoto encyclopedia of genes and genomes
KOBAS: KEGG orthology-based annotation system
LAIR2: Leukocyte-associated immunoglobulin-like receptor 2
MT1M: Metallothionein 1 M
PTEN: Phosphatase and tension homolog
RT-qPCR: Real-time quantitative PCR
TF: Transferrin
TUBB1: Tubulin beta 1 class VI
VEGF: Vascular endothelial growth factor
