# Supplementary material for: The placental transcriptome of the first-trimester placenta is affected by in vitro fertilization and embryo transfer
Source: Reprod Biol Endocrinol. 2019 Jul 1;17:50. doi: 10.1186/s12958-019-0494-7 (PMC6604150; doi:10.1186/s12958-019-0494-7)
Supplement: Supplementary file 1 — Table S1. Detail information on the selection of primers for real-time RT-PCR experiments (DOC 34 kb) [file 12958_2019_494_MOESM1_ESM.doc]

S1 Table. Detail information on the selection of primers for real-time RT-PCR experiments

| Name | Primers sequence (sense) | Primers sequence (antisense) |
| --- | --- | --- |
| AFP | 5`-TCCAGCCAAAGTGAAGAGGG-3` | 5`-CAAGCTGCTITCTCTTAATTC-3` |
| GCM1 | 5`-GGCCGATCCAGCTATATCAA-3’ | 5'-CTGGGGTGCACATAGTGAAA-3` |
| LAIR2 | 5`-GCCATGTCTCCACACCTC -3` | 5'- GAAGTTCACAAGACGGGAGG-3` |
| PTEN | 5`- CGACGGGAAGACAAGACAAGTTCAG-3` | 5'-GCTAGCCTCTGGATTTGACG -3` |
| BCL2 | 5`-CAGATGGCAAATGACCAGCAGA-3` | 5`-TGGCAGGATAGCAGCACAGGAT-3` |
| TF | 5`-GCAATGGGCAGATAGAGTGT-3` | 5`-GTGTTCTTTCGTTCGTGTT-3` |
| MT1G | 5`-TCGCTTGAGATCTCCAGCCTTAC-3` | 5'-ACATCTGGGAGAAGAGCTGTT CC-3` |
| EGFR | 5'-GGACTCTGGATCCCAGAAGGTG-3` | 5`-GCTGGCCATCACGTAGGCTT-3` |
| VEGF | 5`- ATGAACTTTCTCTGCTGTCTTGG-3` | 5'-TCACCGCCTCGGCTTGTCACA-3` |
| TUBB1 | 5`-TTCCAGCTGACCCACTCTCT-3` | 5'- ACAGGGCCTCGTI’ATCAATG-3` |
| GAPDH | 5`-GGTGCTGAGTATGTCGTGGAGT-3` | 5'-CAGTCTTCTGAGTGGCAGTGAT-3` |
