# Supplementary material for: The placental transcriptome of the first-trimester placenta is affected by in vitro fertilization and embryo transfer
Source: Reprod Biol Endocrinol. 2019 Jul 1;17:50. doi: 10.1186/s12958-019-0494-7 (PMC6604150; doi:10.1186/s12958-019-0494-7)
Supplement: Supplementary file 2 — Table S2. The biological processes of up regulated genes in placental subjected to IVF-ET (DOC 68 kb) [file 12958_2019_494_MOESM2_ESM.doc]

S2 Table. The biological processes of up regulated genes in placental subjected to IVF-ET

| No | GO Term | Count | p-Value |
| --- | --- | --- | --- |
| 1 | GO:0006355 regulation of transcription, DNA-dependent | 149 | 1.15×10^-124^ |
| 2 | GO:0006350 transcription | 143 | 1.55×10^-111^ |
| 3 | GO:0055114 oxidation reduction | 82 | 7.98×10^-96^ |
| 4 | GO:0007275 development | 84 | 1.95×10^-49^ |
| 5 | GO:0007155 cell adhesion | 50 | 7.37×10^-43^ |
| 6 | GO:0007165 signal transduction | 91 | 2.55×10^-42^ |
| 7 | GO:0006811 ion transport | 41 | 5.44×10^-33^ |
| 8 | GO:0006468 protein amino acid phosphorylation | 38 | 2.24×10^-30^ |
| 9 | GO:0007399 nervous system development | 39 | 1.39×10^-28^ |
| 10 | GO:0007156 homophilic cell adhesion | 20 | 3.35×10^-25^ |
| 11 | GO:0006508 proteolysis | 39 | 7.31×10^-25^ |
| 12 | GO:0006629 lipid metabolism | 34 | 6.78×10^-24^ |
| 13 | GO:0008285 negative regulation of cell proliferation | 23 | 8.87×10^-23^ |
| 14 | GO:0000122 negative regulation of transcription from RNA polymerase II promoter | 19 | 8.07×10^-21^ |
| 15 | GO:0044419 interspecies interaction between organisms | 22 | 1.55×10^-20^ |
| 16 | GO:0006814 sodium ion transport | 17 | 1.92×10^-20^ |
| 17 | GO:0042632 cholesterol homeostasis | 11 | 1.46×10^-19^ |
| 18 | GO:0006695 cholesterol biosynthesis | 11 | 2.30×10^-19^ |
| 19 | GO:0030154 cell differentiation | 36 | 1.34×10^-18^ |
| 20 | GO:0045944 positive regulation of transcription from RNA polymerase II promoter | 18 | 2.26×10^-17^ |
| 21 | GO:0019941 modification-dependent protein catabolism | 24 | 2.98×10^-17^ |
| 22 | GO:0033344 cholesterol efflux | 8 | 8.82×10^-17^ |
| 23 | GO:0006869 lipid transport | 14 | 5.11×10^-16^ |
| 24 | GO:0008203 cholesterol metabolism | 12 | 2.43×10^-15^ |
| 25 | GO:0006810 transport | 45 | 4.38×10^-15^ |
| 26 | GO:0006953 acute-phase response | 9 | 5.22×10^-15^ |
| 27 | GO:0007596 blood coagulation | 12 | 1.07×10^-14^ |
| 28 | GO:0007186 G-protein coupled receptor protein signaling pathway | 28 | 2.26×10^-14^ |
| 29 | GO:0008202 steroid metabolism | 14 | 9.75×10^-14^ |
| 30 | GO:0030324 lung development | 10 | 1.40×10^-13^ |
| 31 | GO:0006958 complement activation, classical pathway | 8 | 2.78×10^-13^ |
| 32 | GO:0007417 central nervous system development | 16 | 3.78×10^-13^ |
| 33 | GO:0008360 regulation of cell shape | 9 | 4.98×10^-13^ |
| 34 | GO:0006917 induction of apoptosis | 14 | 8.04×10^-13^ |
| 35 | GO:0033700 phospholipid efflux | 6 | 8.93×10^-13^ |
| 36 | GO:0042593 glucose homeostasis | 8 | 1.91×10^-12^ |
| 37 | GO:0006813 potassium ion transport | 12 | 3.47×10^-12^ |
| 38 | GO:0006879 iron ion homeostasis | 7 | 7.83×10^-12^ |
| 39 | GO:0006470 protein amino acid dephosphorylation | 11 | 8.47×10^-12^ |
| 40 | GO:0008652 amino acid biosynthesis | 8 | 1.32×10^-11^ |
| 41 | GO:0006826 iron ion transport | 7 | 1.44×10^-11^ |
| 42 | GO:0005975 carbohydrate metabolism | 18 | 1.44×10^-11^ |
| 43 | GO:0009952 anterior/posterior pattern formation | 10 | 1.55×10^-11^ |
| 44 | GO:0007049 cell cycle | 22 | 2.25×10^-11^ |
| 45 | GO:0006915 apoptosis | 22 | 2.46×10^-11^ |
| 46 | GO:0030168 platelet activation | 7 | 3.26×10^-11^ |
| 47 | GO:0009791 post-embryonic development | 7 | 4.19×10^-11^ |
| 48 | GO:0006641 triacylglycerol metabolism | 7 | 4.19×10^-11^ |
| 49 | GO:0006631 fatty acid metabolism | 12 | 4.63×10^-11^ |
| 50 | GO:0006633 fatty acid biosynthesis | 9 | 5.64×10^-11^ |
