# Supplementary material for: The placental transcriptome of the first-trimester placenta is affected by in vitro fertilization and embryo transfer
Source: Reprod Biol Endocrinol. 2019 Jul 1;17:50. doi: 10.1186/s12958-019-0494-7 (PMC6604150; doi:10.1186/s12958-019-0494-7)
Supplement: Supplementary file 3 — Table S3. The biological processes of down regulated genes in placental subjected to IVF-ET (DOC 70 kb) [file 12958_2019_494_MOESM3_ESM.doc]

S3 Table. The biological processes of down regulated genes in placental subjected to IVF-ET

| No | GO Term | Count | p-Value |
| --- | --- | --- | --- |
| 1 | GO:0006355 regulation of transcription, DNA-dependent | 102 | 8.37×10^-75^ |
| 2 | GO:0007165 signal transduction | 103 | 4.54×10^-58^ |
| 3 | GO:0006350 transcription | 87 | 5.64×10^-55^ |
| 4 | GO:0006508 proteolysis | 53 | 8.85×10^-43^ |
| 5 | GO:0007275 development | 72 | 6.0×10^-42^ |
| 6 | GO:0055114 oxidation reduction | 40 | 5.24×10^-37^ |
| 7 | GO:0007155 cell adhesion | 42 | 1.86×10^-35^ |
| 8 | GO:0007565 pregnancy | 21 | 2.57×10^-34^ |
| 9 | GO:0006468 protein amino acid phosphorylation | 39 | 1.05×10^-33^ |
| 10 | GO:0030154 cell differentiation | 49 | 2.86×10^-33^ |
| 11 | GO:0006916 anti-apoptosis | 26 | 3.89×10^-33^ |
| 12 | GO:0006811 ion transport | 38 | 1.47×10^-31^ |
| 13 | GO:0006915 apoptosis | 36 | 1.92×10^-26^ |
| 14 | GO:0044419 interspecies interaction between organisms | 22 | 9.41×10^-26^ |
| 15 | GO:0006955 immune response | 31 | 6.12×10^-21^ |
| 16 | GO:0008285 negative regulation of cell proliferation | 20 | 6.62×10^-20^ |
| 17 | GO:0045944 positive regulation of transcription from RNA polymerase II promoter | 19 | 9.40×10^-20^ |
| 18 | GO:0006954 inflammatory response | 20 | 7.09×10^-19^ |
| 19 | GO:0006810 transport | 47 | 1.39×10^-18^ |
| 20 | GO:0015031 protein transport | 27 | 1.80×10^-18^ |
| 21 | GO:0006814 sodium ion transport | 15 | 3.26×10^-18^ |
| 22 | GO:0006629 lipid metabolism | 27 | 3.32×10^-18^ |
| 23 | GO:0007399 nervous system development | 27 | 1.13×10^-17^ |
| 24 | GO:0008284 positive regulation of cell proliferation | 18 | 1.00×10^-16^ |
| 25 | GO:0001525 angiogenesis | 14 | 1.07×10^-15^ |
| 26 | GO:0007267 cell-cell signaling | 22 | 1.82×10^-15^ |
| 27 | GO:0006917 induction of apoptosis | 15 | 6.72×10^-15^ |
| 28 | GO:0005975 carbohydrate metabolism | 20 | 1.40×10^-14^ |
| 29 | GO:0006470 protein amino acid dephosphorylation | 12 | 7.20×10^-14^ |
| 30 | GO:0006986 response to unfolded protein | 10 | 8.37×10^-14^ |
| 31 | GO:0007596 blood coagulation | 11 | 8.84×10^-14^ |
| 32 | GO:0006511 ubiquitin-dependent protein catabolism | 14 | 1.29×10^-13^ |
| 33 | GO:0042493 response to drug | 10 | 3.01×10^-13^ |
| 34 | GO:0006457 protein folding | 12 | 1.35×10^-12^ |
| 35 | GO:0007264 small GTPase mediated signal transduction | 17 | 4.94×10^-12^ |
| 36 | GO:0008360 regulation of cell shape | 8 | 9.55×10^-12^ |
| 37 | GO:0007049 cell cycle | 21 | 1.33×10^-11^ |
| 38 | GO:0016192 vesicle-mediated transport | 17 | 1.62×10^-11^ |
| 39 | GO:0045786 negative regulation of progression through cell cycle | 11 | 1.87×10^-11^ |
| 40 | GO:0007229 integrin-mediated signaling pathway | 8 | 2.17×10^-11^ |
| 41 | GO:0006486 protein amino acid glycosylation | 10 | 2.86×10^-11^ |
| 42 | GO:0019941 modification-dependent protein catabolism | 17 | 3.40×10^-11^ |
| 43 | GO:0019221 cytokine and chemokine mediated signaling pathway | 8 | 4.55×10^-11^ |
| 44 | GO:0043123 positive regulation of I-kappaB kinase/NF-kappaB cascade | 9 | 5.65×10^-11^ |
| 45 | GO:0006869 lipid transport | 10 | 6.28×10^-11^ |
| 46 | GO:0007173 epidermal growth factor receptor signaling pathway | 7 | 6.42×10^-11^ |
| 47 | GO:0001558 regulation of cell growth | 10 | 6.77×10^-11^ |
| 48 | GO:0010745 negative regulation of foam cell differentiation | 5 | 6.86×10^-11^ |
| 49 | GO:0007156 homophilic cell adhesion | 10 | 8.45×10^-11^ |
| 50 | GO:0030216 keratinocyte differentiation | 7 | 9.53×10^-11^ |
