# Supplementary figures and images for: The placental transcriptome of the first-trimester placenta is affected by in vitro fertilization and embryo transfer
Source: Reprod Biol Endocrinol. 2019 Jul 1;17:50. doi: 10.1186/s12958-019-0494-7 (PMC6604150; doi:10.1186/s12958-019-0494-7)

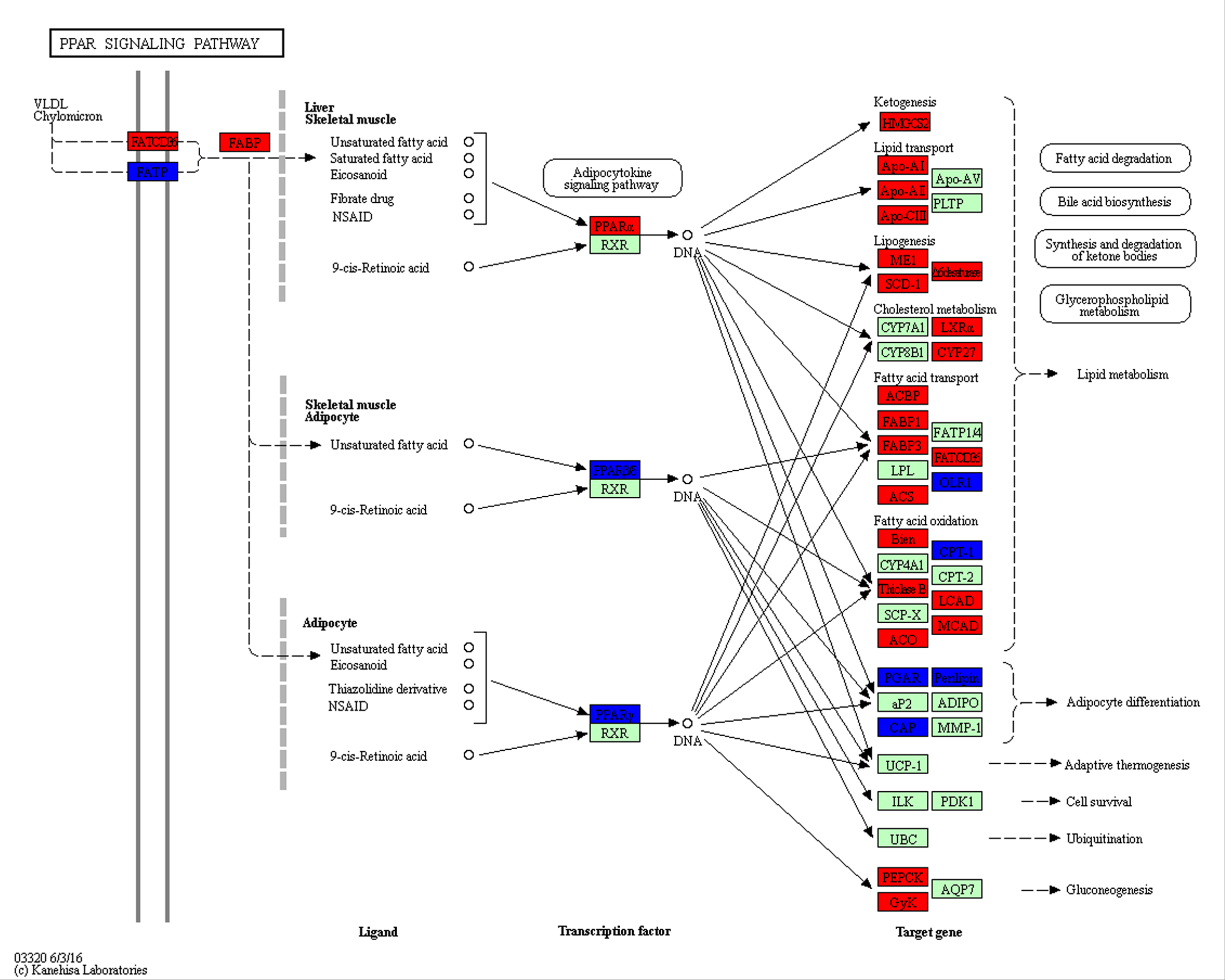

Supplement: Supplementary file 4 — Figure S1. KEGG analysis of the differentially expressed genes, which are labeled, in the PPAR signaling pathway in the placenta after IVF-ET during the first trimester by KEGG. Red, upregulated; Blue, downregulated; Green, no change. (TIF 14811 kb) [file 12958_2019_494_MOESM4_ESM.tif]
